# The maturity index for Uropodina (Acari: Mesostigmata) communities as an indicator of human-caused disturbance in selected forest complexes of Poland

**DOI:** 10.1007/s10493-021-00607-5

**Published:** 2021-04-02

**Authors:** Agnieszka Napierała, Jerzy Błoszyk

**Affiliations:** 1 Department of General Zoology, Faculty of Biology AMU in Poznań, ul. Uniwersytetu Poznańskiego 6, 61-614 Poznań, Poland; 2 The Natural History Collections, Faculty of Biology AMU in Poznań, ul. Uniwersytetu Poznańskiego 6, 61-614 Poznań, Poland

**Keywords:** Bioindicators, Community structure, Environmental monitoring, *K*/*r* reproductive strategy, Natural forest reserve, Soil mites

## Abstract

The necessity of monitoring changes occurring in soil compels us to look for new methods that will allow easy and precise evaluation of the soil quality in a given area. One proposed method is the maturity index (MI) – an index that is based on the distribution of species along the *r*-to-*K* continuum in examined populations. In this study, mites from the suborder Uropodina (Acari: Mesostigmata) were examined for their appropriateness in MI-based assessments of soil quality. The first aim of the study was to establish the criteria on the basis of which the evaluation of the *r*-to-*K* reproductive strategies was conducted for Uropodina in the examined communities. The second aim was to evaluate the performance of the maturity index of communities of Uropodina as indicator of human-caused disturbance in five areas that are legally protected in Poland. The selected areas were: Białowieża Primeval Forest, Gorce National Park, and three nature reserves: Jakubowo, Las Grądowy nad Mogilnicą, and Cisy Staropolskie im. Leona Wyczółkowskiego. We found that as many as 68 out of the 96 analyzed Uropodina species in Poland are *K*-stategists. The highest values of the maturity index were recorded for the nature reserves Cisy Staropolskie im. Leona Wyczółkowskiego and the Białowieża Primeval Forest.

**Supplementary Information:**

The online version contains supplementary material available at 10.1007/s10493-021-00607-5.

## Introduction

Both natural and anthropogenic factors can affect aspects of soil quality that are ecologically and economically important. Many methods have been proposed for assessing and monitoring changes in soil quality. Such methods can be based on faunistic data and community structure of the fauna in the examined area. One index that is based on information about community structure of fauna and life-history traits (i.e., where a species falls on the *r*-to-*K* continuum) is the maturity index (MI) (Ruf 1998; Bongers 1990; Coja and Brucner 2006; N’Dri et al. 2018). This has been used to evaluate the current condition of soil quality, and it was used for the first time by Bongers (1990) with soil nematodes as the focal taxon.

An organism’s life history, much of which involves reproductive strategies, can be viewed as genetically determined traits. Despite the fact that every species has its own reproductive strategy, in the ‘classic approach’, it is thought that there are two extremes of such strategies: *r* and *K* (see, e.g., MacArthur and Wilson 1967; Pianka 1981, 2011; Krebs 2011). MacArthur and Wilson (1967) associate *r-*selection with typically seasonal and unstable habitats in temperate climates, where resources are seasonally available in large quantities, resulting in high reproduction rates. Species that fall towards the more ‘*r*-selected’ end of the continuum are often referred to as ‘opportunistic’ species. Because ‘*r*-selected’ species often occupy regions with unstable climate or environmental conditions, local extinction and recolonization can frequently occur (MacArthur and Wilson 1967). The species that fall towards the ‘*K-*selected’ end of the continuum live in more stable and predictable environments. Their populations fluctuate around the carrying capacity, and extinction and recolonization occur less frequently. They produce fewer offspring but spend more resources per offspring, which have a higher probability to survive into adulthood. ‘*K-*selected’ species are frequently described as being at ‘equilibrium’ in the literature (MacArthur and Wilson 1967; Pianka 1981, 2011; Krebs 2011).

The ‘*r*-*K*’ selection theory was popular in the 1970s and 1980s (Blute 2016), but in the early 1990s it lost importance because it was criticized for disparity between the theoretical concepts and empirical studies (e.g., Stearns 1992; Roff 1993). The main problems were the oversimplification of life history strategies of organisms and the fact that many species do not match the classification (Stearns 1992). Pianka (1970) pointed out that no organism can be definitely classified as either an ‘*r*- or *K* strategy’ organism. He described the *r*-*K* strategies as a ‘continuum’, in which different species can be placed relative to the two extremes. The main criterion to establish the actual strategy of a species may be applied if a great deal is known about an organism and its environment (Reznick et al. 2002). This approach is most common in contemporary ecology and evolutionary biology (Reznick et al. 2002; Reynolds 2003; Dodson 2007).

Organisms that can serve as bioindicators include soil mites (Kimberling et al. 2001; Gulvik 2007; Ivan and Vasiliu 2009; Aspetti et al. 2010; Andrievskii and Syso 2012; Meehan et al. 2019). At the assemblage level, soil mite communities have frequently been shown to be sensitive to changes in environmental conditions and habitat disturbance, resulting in changes in the species composition and abundance (see also Napierała 2008; Napierała et al. 2014). For this reason, they can be quite useful as bioindicators (Kay et al. 1999; Koehler and Melecis 2010; Napierała et al. 2014). Research that first used the maturity index of soil mites concerned predatory soil mites from the suborder Gamasina (Mesostigmata) (Ruf 1998). The author used the gamasine MI to evaluate the environmental impact of pollution on forest soil in Germany (Ruf 1998). Similar research was carried out on communities of gamasine mites to evaluate soil quality in five natural forest stands in Austria (Coja and Brucner 2006). Another study was carried out in rubber plantations of different ages in Ivory Coast and the maturity index was used as an indicator of environmental change (N’Dri et al. 2018). These studies relied mainly on coarse taxonomic units, that is, families.

As has been rightly pointed out by N’Dri et al. (2018), the traditional way of evaluating soil quality, based only on physical and chemical criteria, does not allow to state with certainty which environmental conditions are favorable or unfavorable for soil mesofauna. The maturity index (Ruf 1998; Coja and Brucner 2006; N’Dri et al. 2018), based on ranking mites according to their life-strategy *r*/*K* traits, can be used as a supplementary method in evaluation of soil quality in forest ecosystems. As opposed to species of Gamasina, especially those with an *r*-strategy, which are more common in areas transformed by humans (Eisenbeis 2006), Uropodina mites are rare in such places (Błoszyk 1999; Napierała 2008; Napierała et al. 2014, 2018) because they have very specific environmental requirements (Błoszyk 1999; Błoszyk et al. 2003, 2004; Napierała 2008; Napierała et al. 2014).

The current study is intended to provide an evaluation of the degree of human disturbance in five legally protected areas in Poland. Areas with low human disturbance are defined here with a number of habitat characteristics which are favorable for maintaining and preserving high biodiversity, i.e. the more species there are in the studied communities, especially rare and stenotopic, the lower human impact has been in such areas. The presented evaluation is based on an analysis of material collected from communities of soil mites from the suborder Uropodina (Mesostigmata), including calculations of the maturity index. Uropodina are among one of the best-known groups of soil mites in Europe with more than 440 described species (Wiśniewski and Hirschmann 1993). In Poland there are between 137 and 150 species of these mites (Wiśniewski 1997; Błoszyk 1999, 2008). Most Uropodina are steno- and oligotopic species (Błoszyk 1999; Błoszyk et al. 2003, 2004), with very specific environmental requirements. These mites are very sensitive to changes in the conditions of the environment. For example, decrease of the level of groundwater can result in a decrease or entire loss of the hygrophilous species (Błoszyk 1999; Napierała 2008; Napierała et al. 2014). Also, pollutants such as acid rains and gradual deposition of different pollutions in the soil eliminate the more fragile species, such as *Trachytes lamda* Berlese and *Trachytes minima* (Kramer) (Błoszyk 1999). Uropodina inhabit soil and litter of different types of forests as well as open areas (meadows, xerophilous grasses, sedgelands, sandhills, agrocenoses etc.) (Athias-Binche 1981a,b,c; Błoszyk 1999; Mašán 2001; Błoszyk et al. 2003; Napierała et al. 2009) and ephemeral microhabitats (merocenoses), such as rotten wood, anthills, termite nests, nests of birds and small mammals, and animal excrement (e.g., Błoszyk 1999; Mašán 2001; Błoszyk et al. 2003; Napierała and Błoszyk 2013; Napierała et al. 2016). There are different types of life history strategies in this group. The species that inhabit soil and litter are often parthenogenetic (thelytokous), whereas those that occur in merocenoses are mostly sexual (e.g., Błoszyk 1999; Mašan 2001; Błoszyk et al. 2003, 2004; Napierała and Błoszyk 2013; Napierała et al. 2016). Many uropodines disperse by mean of phoresy (Błoszyk 1999; Gwiazdowicz 2000; Bajerlein and Błoszyk 2004). More detailed accounts of Uropodina as bioindicators used to evaluate the condition of the environment can be found in many earlier studies (Błoszyk 1999; Błoszyk et al. 2003, 2004; Napierała 2008; Napierała et al. 2016, 2018).

The major aim of this study was to propose and test a range of criteria to characterize the life history strategies (focusing on reproductive strategies) of Uropodina species. This in turn allowed us to assign locations on the *r*/*K* scale to uropodine mites occurring in Poland. The second aim of the study was to use the maturity index (MI) method for Uropodina communities as an indicator of human disturbance in five legally protected areas in Poland. In the case of soil fauna, including Uropodina, there is no single factor which had a direct impact on species diversity of communities, and that could be used as a simple, measureable and objective estimate of soil quality, which is very important for the biodiversity in a given area (N’Dri et al. 2018). Instead, there is a set of factors, some of them still unknown, that determine differences in biodiversity between different areas. In many previous studies (Błoszyk 1999; Napierała 2008; Napierała et al. 2009, 2018, 2020), we showed that high biodiversity of Uropodina in forests is strongly correlated with such factors as the tree stand age and species composition, size and legal status of the area (whether the area is legally protected and how long it has been protected), and the degree of human-caused disturbance. The areas selected for this study are different in their legal status (nature reserves or national parks). In Poland, national parks are the strictest form of legal protection of nature. A national park covers areas of a minimum of 1000 ha. Besides being larger than nature reserves, another difference is that whole ecosystems are protected in national parks, and the large area of the park is tantamount to higher biodiversity of the protected habitats. Moreover, national parks can contain strict nature reserves, with no human interference. In nature reserves, habitats and ecosystems are preserved in their natural form or with minor modifications, usually with unique natural value. The most relevant differences between the objects selected for this study, were the size of the area they cover, the period they have been legally protected, their geographical location, and the degree of human-caused disturbance.

It is worth mentioning that detailed information on the habitat in the analyzed areas is not always available. For this reason, we think that, the maturity index can be used to evaluate the degree of human-caused disturbance of an area when only faunistic information is available. Thus, our aim was to test the hypothesis that the maturity index will be highest for areas where the degree of human-caused disturbance was lowest, and therefore in places with the oldest and most natural tree stands, which have been legally protected for a very long time. This in turn will allow us to check whether the maturity index can be used as an index of the natural value of areas.

## Materials and methods

The research was conducted in five areas located in different regions of Poland: Białowieża Primeval Forest, Gorce National Park, and three nature reserves: Jakubowo, Las Grądowy nad Mogilnicą, Cisy Staropolskie im. Leona Wyczółkowskiego (Table 1).

**Table 1:** Location, legal status, and number of collected samples in the examined areas

Area	Białowieża primeval forest*	Gorce national park	Jakubowo nature reserve	Las Grądowy nad Mogilnicą Nature Reserve	Cisy Staropolskie im. Leona Wyczółkowskiego Nature Reserve
Location	Northern-East Poland, 52°46′N 23°52′E	Southern Poland, 49°33′N 20°06′E	Western Poland, 52°29′N 16°16′E	Western Poland, 52°28′N 16°14′E	Northern-west Poland, 53°30′N 18°07′E
Legal status	National park, strict nature reserve and unprotected areas	National park	Nature reserve	Nature reserve	Nature reserve
Foundation year	1932	1981	1959	1959	1827
Age of tree stand [years]	> 150	100–120	> 150	< 100	> 150
Area[ha]	10,517	7030	4.02	7.32	113.61
Number of qualitative and quantitative samples	439	824	1561	399	452
References	Napierała et al. 2020	Błoszyk and Napierała 2018	Błoszyk and Napierała 2018	Błoszyk and Napierała 2018	Błoszyk and Napierała 2018

*Białowieża National Park nature reserve ‘Lasy Naturalne Puszczy Białowieskiej’ and the economic areas of the Białowieża Primeval Forest

Mites were extracted from soil samples collected with both qualitative and quantitative methods. The quantitative samples were collected with a cylinder of 5 cm diameter to the depth of 10 cm. The qualitative samples (about 0.5–0.8 l) consisted of sieved litter and soil, collected in the five research areas. The use of both types of samples allowed us to obtain much more information about the actual species composition of the communities in the examined areas (Błoszyk and Napierała 2018). For our analyses we used data about uropodine assemblages published in earlier studies. In all areas soil was slightly alkaline with mull humus. Table 1 shows the most important information about the examined areas with references to studies containing detailed descriptions of the areas and detailed sampling methods.

Based on earlier studies, we used the following classes of ecological indices for Dominance (D%) and Frequency (C%) (Kasprzak and Niedbała 1981; Błoszyk 1999): Dominance: D5, eudominants (> 30% of uropodine individuals); D4, dominants (15.1–30.0%); D3, subdominants (7.1–15.0%); D2, residents (3.0–7.0%); and D1, subresidents (< 3%). Frequency: C5, euconstants (present in > 50% of samples); C4, constants (30.1–50%); C3, subconstants (15.1–30.0%); C2, accessory species (5.0–15.0%); and C1, accidenal (< 5%).

The Shannon Wiener diversity index (H’) was calculated for uropodine assemblages in each area.

### Criteria for classification of Uropodine species according to the r and K strategies

It should be borne in mind that determining the correct position of a species on the *r*-to-*K* continuum for such tiny organisms as soil mites, about which we still know very little, is extremely difficult and it is possible only for some groups. We are fully aware of the difficulties in working out the correct life-history classification of such small organisms as soil mites, especially due to the fact that we still have to learn much about these organisms, and what we already know may turn out insufficient to ascertain precisely their life history strategies. However, this does not hold for the species studied here; long-term research into Uropodina in Poland allows us to assign the species known from this country to relative categories on the *r*-to-*K* continuum of life strategies. In this study we have adopted the standard interpretation of the *K* and *r* strategies, and following Bongers (1990) and Ruf (1998), we decided to perceive *r-*strategists as better ‘colonizers’, that is, species whose characteristics allow them to colonize areas very fast under favorable conditions. Such species are therefore most abundant and frequent in samples of disturbed habitats, and they are characterized by higher ecological tolerance (eurybions, polybions), which allows them to occur in different types of environment, including those modified by humans. Besides these, there are also species with reproduction capabilities that allow rapid population growth as well as better dispersal capabilities for example by means of phoresy. *K*-strategists (see Bongers 1990) never belong to dominant species because they have a narrow range of ecological tolerance (stenobionts, oligobionts). They reproduce slower, which results in a lower population growth rate. *K*-strategists also have lower dispersal capabilities.

On the basis of data collected in the period 1961–2017, stored in the digital database of the Natural History Collections at the Faculty of Biology at Adam Mickiewicz University in Poznań, and the information from many earlier studies (Athias-Binche 1981a,b,c; Wiśniewski 1997; Błoszyk 1999; Mašán 2001; Błoszyk et al. 2003, 2004), on the biology, ecology, and geographical distribution of Uropodina in Poland, we have classified each uropodine species according to their life-history strategies. Thus, we decided that the most significant characteristics for determining the type of life strategy for each species of Uropodina are as follows: abundance expressed through dominance (D) and frequency (F), range of ecological tolerance (range of occupied habitats), population growth rate, and phoretic capabilities of the species (Tables 2, 3).

**Table 2:** Particular criteria for determining life strategy of Uropodina

	*r-*Selection	*K*-Selection
Ecological indices (dominance and frequency)	High	Low
Ecological tolerance based on habitat breadth	More tolerant (P, E)*	Less tolerant (S, O)*
Population growth rate	High	Low
Occurrence of larvae	Larvae present almost for the whole year	Larvae present during several months
Phoresy	When numerous deutonymphs are carried (effective phoresy) or lack of phoresy (in the case of parthenogenetic soil species)	When very few deutonymphs are carried

*E eurybionts, *P* polybionts, *O* oligobionts, *S* stenobionts, adopted from Błoszyk et al. (2004) and Napierała et al. (2018)

**Table 3:** Number of points for particular criteria

Dominance (D)	Frequency (F)	Ecological tolerance (H)*	Population growth rate (R)***	Occurrence of larvae (L)	Phoresy (Ph)
D1 – **5**	C1 – **5**	Stenobionts – **4**	Low– **1**	Larvae present during 10–12 months/year – **0**	Effective phoresy or phoresy not present – **0**
D2 – **4**	C2 – **4**	Oligobionts – **3**	High – **0**	Larvae present during 9–6 months/year – **1**	Less effective phoresy – **1**
D3 – **3**	C3 – **3**	Polybionts – **2**		Larvae present 5 months/year or less, or larvae not found ** – **2**	
D4 – **2**	C4 – **2**	Eurybionts – **1**			
D5 – **1**	C5 – **1**				

*The ecological tolerance (H), fertility strategy (Fs) have been adopted from Błoszyk et al. (2004) and Napierała et al. (2018)

**No larvae were found in the material in the case of very rare species

The species were ranked on a numerical *r*/*K*-scale (Table S1) on the basis of a set of characteristics. Every characteristic such as abundance, frequency, range of ecological tolerance, dispersal capabilities and population growth rate, has its own range of points (Table 3). In general, categories *K*1 to *K*3 have been given to species which had low abundance and were very rare (i.e. they had low dominance and frequency) in the examined communities, and that also had narrow habitat preferences (i.e. they were stenotopic and oligotopic species) (Tables 2, 3). In the case of Uropodina, species with low ecological tolerance often occur in specific ephemeral microhabitats (Błoszyk 1999; Napierała and Błoszyk 2013) and in rare habitats, including those disappearing and endangered, such as old forests, xerophilous grasses, rocks grasses, peat-bogs (Błoszyk 1999; Błoszyk et al. 2003). Categories *r*1 to *r*3 were assigned to species that were most abundant in the samples (i.e. they had the highest values for the indices of dominance and frequency). Such species occurred in many different habitats (i.e. they were polytopic and eurytopic species), including areas with a high degree of human-caused disturbance and have high population growth rates.

Uropodina species with high population growth rates are mostly common soil-dwelling species, which reproduce by means of thelytoky (Błoszyk et al. 2004). About 27% of the Uropodina species known from Poland were identified as reproducing only (or almost exclusively) parthenogenetically, but these species are most dominant in communities inhabiting soil (Błoszyk 1999; Błoszyk et al. 2004). Different studies show that thelytokous species of Uropodina are more dominant in communities in northern areas, for example, in Norway, and their percentage in communities is lower in southern areas (Wiśniewski 1993; Błoszyk 1999; Mašan 2001; Błoszyk et al. 2003). This mode of reproduction allows them to colonize and increase in densities under favorable soil conditions faster than do sexually reproducing species. For this reason, we classified most Uropodina species with thelytokous reproduction capabilities as *r*-strategists with high population growth rates. The only exceptions in this group are two species from the genus *Oodinychus*: *Oodinychus ovalis* (CL Koch) and *Oodinychus karawaiewi* (Berlese), which are common and abundant in the soil (Błoszyk 1999; Błoszyk et al. 2003, 2004; Napierała 2008; Napierała et al. 2009), and often occur in microhabitats such as dead wood, tree holes, mammals and birds nests (Napierała and Błoszyk 2013), though, they reproduce sexually. *Oodinychus karawaiewi* is regarded as a synanthropic species because it dominates in soil communities of areas with evident characteristics of human-caused disturbance, such as parks, shrubs and economic forests (Błoszyk et al. 2006).

The results of long-term research (Błoszyk and Olszanowski 1985; Błoszyk 1999; Błoszyk et al. – in press) also show that larvae of the most abundant and common species of Uropodina, such as *T. aegrota*, *T. pauperior*, *O. ovalis*, *U. tecta*, *O. minima* (Table S1) occurred in the collected samples for most of the year (10–12 months), and the highest abundance was usually recorded at the beginning of summer, when the temperature of soil was also highest (Błoszyk 1999, Błoszyk et al. – in press). The results of previous studies show that seasonal fluctuations in temperature is one of the main factors affecting the frequency of laying and hatching of eggs in Uropodina mites (Błoszyk and Olszanowski 1985; Błoszyk 1999). In the case of less abundant, but still fairly common species (e.g., *D. perforatus*, *D. carinatus*, *C. cassideasimilis*, etc.) (Table S1), larvae were recorded in samples less frequently (< 6 months) and their abundance was fairly consistent when they did occur.

The second group of Uropodina comprises species with low population growth rates (Table S1). Most species in this group reproduce sexually, and their populations consist of both males and females (Błoszyk 1999; Błoszyk et al. 2004). This characteristic is typical for species that are not very common or even rare because they are associated with particular conditions in ephemeral microhabitats (Napierała, Błoszyk 2013). When the environmental conditions change and the microhabitat disappears, these species migrate (by means of phoresy). This group comprises also rare and very rare soil-inhabiting thelytokous species (e.g., *Trachytes lamda*, *T. minima*, *T. montana*, *Cilliba cassideasimilis,* etc.) (Table S1). Moreover, these species are characterized by the lowest number and occurrence of larvae throughout the year (in some cases no larvae were found because the species were very rare). The low reproduction rate was regarded as a characteristic of *K*-strategy species.

As for the dispersal capabilities of Uropodina, the phenomenon of phoresy seems to be extremely important in colonization of new, ephemeral island-like habitats (see eg. Faasch 1967; Athias-Binche and Habersaat 1988; Wiśniewski and Hirschmann 1992; Athias-Binche 1993; Athias-Binche et al. 1993; Mašán 1993, 1994; Błoszyk 1999; Gwiazdowicz 2000; Bajerlein and Błoszyk 2004, Napierała et al. 2015). Generally speaking, phoretic species are more expansive and widespread. The results of our long-term research (Błoszyk 1999; Bajerlein and Błoszyk 2004, Gwiazdowicz et al. 2013; Napierała and Błoszyk 2013; Napierała et al. 2015, 2016; Konwerski et al. 2017, 2019; unpublished data) show that phoresy is a phenomenon which can occur with a different pace of intensity. That means that the number of phoretic deutonymphs on a host differs among different uropodine species and also the intensity of phoresy during the whole year can be different for particular species (Konwerski et al. 2017,2019). We have observed that in some species (e.g., *U. orbicularis*, *P. pulchella, O. ovalis*, *P. patavinus*, *L. orbicularis*, *P. borealis, T. sociata, T. polytricha, A. infirmus*) the number of carried deutonymphs was very high, whereas it was low in others (e.g., *M. paradoxa*, *U. nova, T. bipilis, P. pyriformis*). Production of large numbers of phoretic deutonymphs in some species considerably increases their dispersal capabilities (Bajerlein and Błoszyk 2004; Konwerski et al. 2017, 2019). A large number of deutonymphs carried by a host is very important for species which reproduce sexually, as it considerably increases the possibility of finding a mate. This ability is also important for parthenogenetic species because a high number of deutonymphs carried into a new habitat can give rise to a more numerous population. For these reasons, phoretic species were considered as *r*-strategists, but only those species that exhibited so-called ‘effective’ phoresy, i.e. when the vector carries a large number of deutonymphs (Tables 2, 3, S1). Species with ‘less effective’ phoresy, i.e. where vectors carried a few deutonymphs, were classified as *K*-strategists (Table 3, S1).

We recognize three separate categories within each type of strategy (*r*1–*r*3, *K*1–*K*3) (Table S1). The summed value of the criteria mentioned above (Table 2) stands for the following categories on the *r* to *K* continuum:

*r*-categories: *r*1 (3–10), *r*2 (11–14), *r*3 (15).

*K*-categories: *K*1(16), *K*2 (17), *K*3 (18).

### Maturity index for Uropodina mites

After determining the location on the *r*-to-*K* scale of the analyzed Uropodina (Table S1), we calculated the maturity index (proposed by Ruf 1998 and N’Dri et al. 2018) for each community in the examined areas (Table S2). The maturity index for communities of Uropodina is calculated as the weighted proportion of *K*-selected species in the whole community of Uropodina. The value of the index should be higher in less environmentally disturbed areas. The minimum value of the maturity index is zero (no *K* strategists in the site), and the maximum value is 1 (all species are *K* strategists) (N’Dri et al. 2018). The maturity index is given by:

$$MI=\frac{\sum_{i=1}^{S}Ki}{\sum_{i=1}^{S}Ki+\sum_{i=1}^{S}ri}$$

with S – number of species, *K* – *K-*value ranging from 1 to 3, *r* – *r*-value ranging from 1 to 3 for the species i.

## Results

### r-to-K rankings of the analyzed Uropodina

**Fig. 1:**
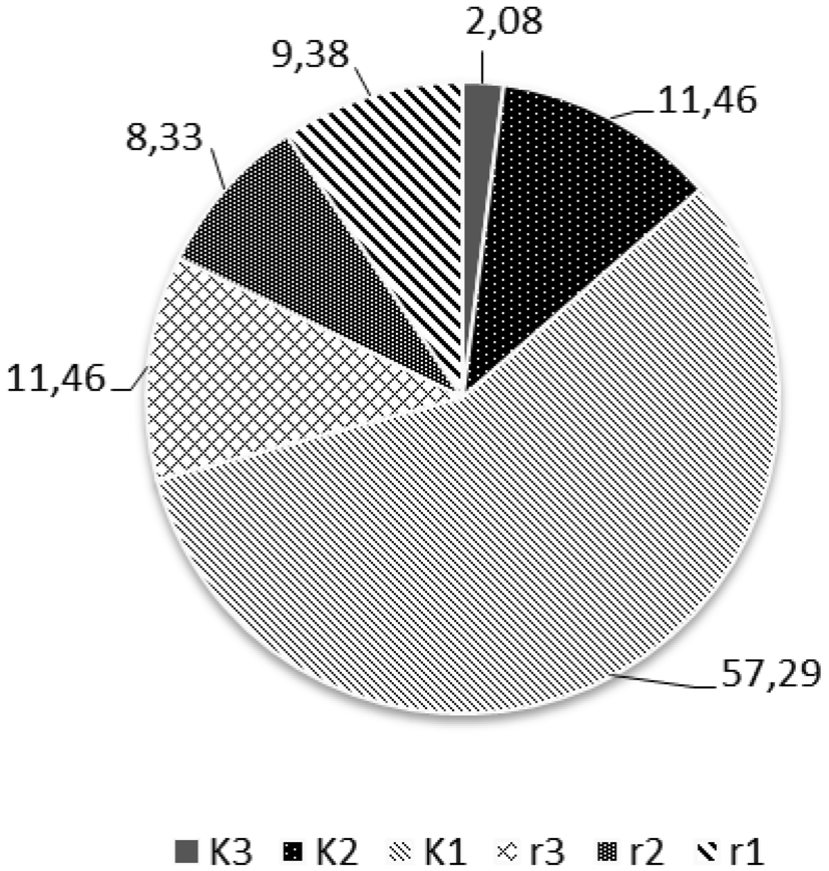
Percentage of species with different *K* and *r* strategies among Uropodina in Poland

Out of the 96 species analyzed in this study, 68 species were classified as *K*-strategists (Table S1; Fig. 1); 13 species were assigned to categories *K*3 and *K*2, indicating that they were rare and very rare stenotopic species. Twenty eight species of Uropodina found so far in Poland were classified as *r*-species. In total, only 9% of the 96 species were very common and abundant, with a high rate of reproduction (*r*1-species) (Fig. 1).

### Evaluation of habitat quality measured with the maturity index in selected forest complexes in Poland

In the examined areas we found a total of 43 species of Uropodina (Table S2). The highest number of species was recorded in the nature reserve Cisy Staropolskie im. Leona Wyczółkowskiego and in the area of the Białowieża Primeval Forest (72.1 and 53.5%, respectively, of the 43 species). These areas also had the highest values of the Shannon Wiener diversity index (Table 4). The lowest number of uropodine mite species was recorded for the two nature reserves Jakubowo NR and Las Grądowy NR (i.e. 15 and 11 species).

**Table 4:** The values of the maturity index (MI) for the examined areas and percentage of r-species in the communities. H’ - Shannon Wiener diversity index

	Cisy Staropolskie im. Leona Wyczółkowskiego NR	Puszcza Białowieska	Gorce National Park	Jakubowo NR	Las Grądowy nad Mogilnicą NR
% of K-species	41.94	34.79	16.67	33.33	18.18
MI	0.32	0.31	0.11	0.27	0.21
H’	2.060	2.807	1.315	1.587	1.589

**Fig. 2:**
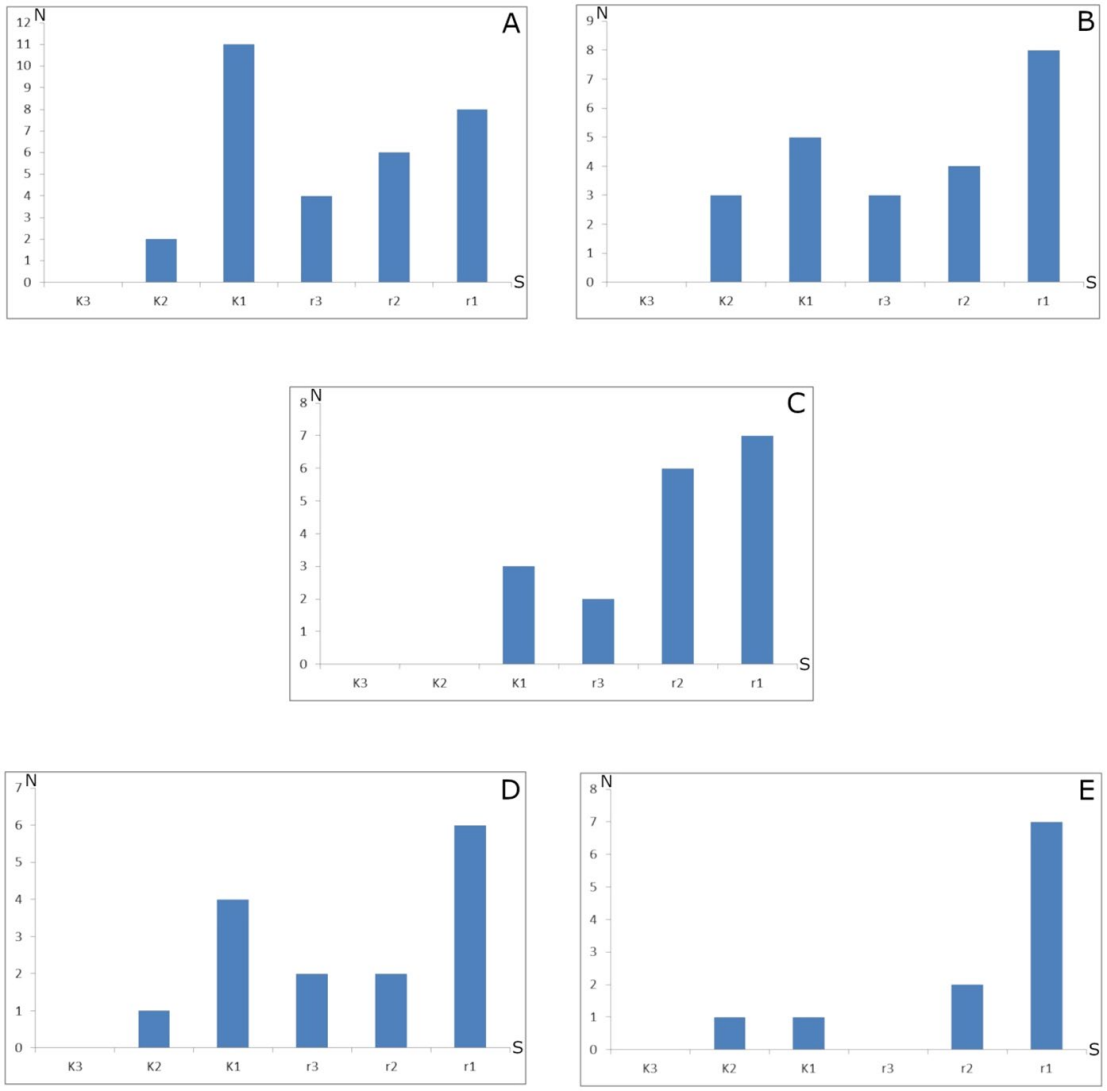
The number of Uropodina species (N) and their reproductive strategies (S) in the examined areas. **a** Cisy Staropolskie im. Leona Wyczółkowskiego Nature Reserve, **b** Białowieża Primeval Forest, **c** Gorce National Park, **d** Jakubowo Nature Reserve, **e** Las Grądowy Nature Reserve

The highest values of the maturity index was recorded for the Cisy Staropolskie im. Leona Wyczółkowskiego NR (0.32) and in the Białowieża Primeval Forest (0.31) (Table 4). The lowest values of the maturity index were calculated for the material collected in the Las Grądowy nad Mogilnicą NR (0.21) and in Gorce National Park (0.11). In all examined areas, *r*-strategy species prevailed. Representation of *K*-strategy species was the highest in Cisy Staropolskie im. Leona Wyczółkowskiego NR (42%) and the lowest in Gorce National Park (17%) (Table 4). The number of *K2* and *K1* species was the highest in the reserve Cisy Staropolskie im. Leona Wyczółkowskiego NR and in the Białowieża Primeval Forest (Fig. 2). *K*3 species were not found in any of the studied areas. In Gorce NP species from category *K*2 were not found. Moreover, *r*3-species were not detected in the material from Las Grądowy NR.

## Discussion

The research carried out into Uropodina for over 50 years (Athias–Binche 1977a,b,c, 1981a,b,c, 1982a,b,^1983^; Błoszyk 1984, 1985, 1999; Wiśniewski and Hirschmann 1993; Wiśniewski 1997; Mašán 2001; Błoszyk et al. 2003), has provided a lot of information about the biology, ecology and geographical distribution this group of mites. These allow to obtain valuable information which can be used to estimate the location on the *r*-to-*K* continuum of particular uropodine species. In the analysis presented in this study we have used both the information about the biological characteristics of the species, i.e. the reproductive strategies, dispersal abilities (e.g., phoresy), and about their ecology regarding the habitat preferences, abundance, frequency, and conservation status. Because the biology of Uropodina in Poland is very well studied, we were able to apply the maturity index approach. The results of the analysis presented in this study show that almost ¾ (68 species) out of all analyzed Uropodina species (Table S1) were *K*-strategy species, and hence, they can be used as bioindicators to assess soil quality in natural areas. This can be seen in the study published by N’Dri et al. (2018), who classified the family Uropodidae as taxon with life history trait class *K*3.

In our opinion, the use of Mesostigmata mites at the level of family for calculations of the maturity index is problematic. Undoubtedly, it has a few advantages, as it allows to easily evaluate the components of a community as to their life strategies without the need to identify them to the species level, and does not require expert knowledge (N’Dri et al. 2018). However, it also has some drawbacks. Assigning all species of a group with hundreds of species with different life-histories to one category in the *r*-*K* continuum is an oversimplification in our opinion. Even within one genus, species are sometimes characterized by different reproductive strategies, ecological tolerance and frequency of occurrence. A good example of such species of Uropodina are those from the genus *Trachytes* or *Olodiscus*. For example, *T. aegrota* is a thelytokous, eurytopic, and common species, with broad occurrence, whereas *T. lamda* is a very rare stenotopic, thelytokous species, which prefers old deciduous tree stands, and *T. minima* is a very rare sexual stenotopic species (Carpathian endemic species), with a narrow range of occurrence. Other examples include also such species as *T. montana* – a thelytokous, rare, mountainous species with narrow range of occurrence, *T. irenae* – a common species reproducing sexually with narrow range of occurrence. As for the genus *Olodiscus*, *O. minima* is a common thelytokous eurytopic species. *Olodiscus misella* is a rare thelytokous species with narrow range of occurrence associated with beech trees, and *O. kargi* is a very rare sexual species. This is why, in our opinion, the finer the taxonomic status of the group used as a bioindicator, the more reliable the obtained results will be in the analysis. This principle is also applicable to the whole spectrum of ecological research, which often focuses on communities of invertebrates. The use of taxa identified at the higher levels is prone to give only a general overview, and the obtained results will therefore be less reliable. Nevertheless, such studies also provide valuable information and they allow to assess soil health or the extent of pollution (see, e.g., Bongers 1990; Ruf 1998; Manwaring et al. 2018; N’Dri et al. 2018; Meehan et al. 2019). However, in this case we had a large set of data collected during long-term research revealing a lot of information about the biology and ecology of the discussed group of mites, and therefore, in this study the analysis is based on data at the species level.

Our calculations of the maturity index based on Uropodina revealed that the most valuable place from the point of view of these mites, is the Cisy Staropolskie im. Leona Wyczółkowskiego NR. This is the oldest nature reserve in Poland (Table 1) (and one of the oldest reserves in Europe), which, similarly to the Białowieża Primeval Forest, is characterized by high species richness of Uropodina communities and high percentage of *K*-selected species. This reserve is in fact the largest area with the common yew (*Taxus baccata*) in Europe, with unique habitat, where the conditions for mites from the suborder Uropodina are very good. The long period of legal protection and isolation have resulted in very high species diversity of Uropodina, which in some cases is similar or even higher than in some national parks with much larger areas and with higher overall biodiversity.

The results obtained show that the second maturity index was for the Białowieża Primeval Forest, which is extremely valuable with old and unique tree stands (Table 1). However, the calculated value of maturity index was the second (0.31, Table 4), probably because almost half of the analyzed material came from a part of the Białowieża Primeval Forest that is not legally protected. The high biodiversity of habitats in this area caused the high number of the species found in the soil (23) (Table S2), and the number of *K*-selected species was also high (Table S2; Fig. 2), and they were rare species with very specific habitat preferences. Also the Shannon Wiener diversity index for the examined community was the highest (Table 4). The Białowieża Primeval Forest is unique in Europe, and thus it deserves special legal protection, as it is one of the very last places in Europe where researchers can observe natural processes with no or very little human-caused disturbance (Kujawa et al. 2016; Lachat and Müller 2018). Nowadays, only 17% of about 62,000 ha of the Polish part of the Białowieża Primeval Forest is legally protected as a national park, about 19% is regarded as nature reserve, and the other parts of this unique forest complex are economic forests (Pracownia na rzecz Wszystkich Istot 2019; WWF.pl 2019). Although the whole Belarussian part of the Białowieża Primeval Forest is legally protected as a national park, in Poland only half of it is legally protected. According to the data from various publications since the 1960’s, 60 species of Uropodina have been recorded from the Polish part of the forest so far (Napierała et al. 2020), which is ~ 40% of the entire uropodine fauna of Poland. This strongly suggests that to effectively protect habitats of both Uropodina and potentially many other groups of organisms, it is necessary to legally protect larger areas of this forest complex.

The third area where the maturity index suggests that the soil environment is in a very good condition for uropodine mites is Jakubowo. Despite the fact that this reserve is fairly small (4.02 ha of legally protected area), it still has one of the most beautiful fragments of old oak-hornbeam forest (ca. 200 years old) (Table 1) with its beech-variant (*Galio sylvatici-Carpinetum* var. with European beech, *Fagus silvatica*), and it offers very good conditions for Uropodina mites (Błoszyk 1999; Napierała 2008; Napierała et al. 2009). The high mite richness in this reserve had been also observed in many earlier studies (Błoszyk 1999; Napierała 2008; Napierała et al. 2009, 2014), and the evaluation of soil habitat quality in this place with maturity index supports the earlier results.

The last two legally protected areas, where the maturity index values are lowest, are Las Grądowy nad Mogilnicą NR and Gorce National Park. The first one is a small nature reserve established in the same year as the neighboring Jakubowo NR to protect a part of a larger hornbeam forest. Tree stands in this reserve are younger than in Jakubowo NR (Table 1) and the area is under high human-caused disturbance due to roads and unpaved footpaths and cutting down of trees. The uropodine community in this area consists mainly of common *r*-species (Fig. 2). The only exceptions in this respect was one *K*2 species – *T. lamda*, which is indeed rare and sensitive to changes in soil environment (Błoszyk 1999). This species has not been recorded from this reserve after 2000 (Napierała 2008; Napierała et al. 2014), which suggests that the soil habitat quality in this reserve has been gradually deteriorating since then (Napierała 2008; Napierała et al. 2014). The lowest values of the number of species and the maturity index have been recorded in the case of Gorce National Park. This is a relatively young national park, which was established in 1981 (Table 1). The number of uropodine species found in the material from this park is fairly high (18). However, there were no *K*2 and *K*3 species (Fig. 2), and *r*1 and *r*2 species were most abundant, which means that the community in this area consists mainly of common eurytopic species of Uropodina.

When we compare the values of the Shannon Wiener diversity index with the maturity index values (Table 4) for each area, the results are in general similar. However, we suggest that the Shannon–Wiener index is less accurate because it gives similar results for communities with a similar number of species and abundance. It treats each species the same, as it does not take such characteristics of species as their biology, rarity, ecological demands, etc. into account. A good example of this can be seen for Jakubowo NR and Las Grądowy nad Mogilnicą NR, where the number of species is similar, the reserves are closely located, and their tree stands are also similar, but the values of the maturity index calculated for these reserves are different.

Species composition of soil mesofauna in a given area is the product of many different factors, such as the history of the area, diversity of the existing habitats, natural value and geographical location, which will have bearing on the presence or lack of certain species. With regard to Uropodina in Poland some species, e.g., those inhabiting Eastern Carpathians, will be present only in the southern parts of Poland. The fact that these mites have not been found in the material analyzed here stems from the geographical distribution of the species and their ecological requirements, and not from the lower natural value of the examined area. If one wants the maturity index to be a reliable indicator of soil quality or natural value of a given area, then the fauna of the region must be well known and all criteria enumerated above must be taken into account.

Uropodina mites are one of the many components of the soil mesofauna and their presence, species composition and abundance in communities reflect certain characteristics of the forest areas in which they occur. This has been observed in many earlier studies (Błoszyk 1999; Napierała 2008; Napierała et al. 2009, 2014, 2018). In this study these correlations have been confirmed by the highest values of maturity index in areas with the oldest tree stand and longest strict legal protection status, where the degree of human-caused disturbance is very low. Our analysis shows that the maturity index is a more sophisticated tool of evaluation of the degree of human disturbance than the Shannon Wiener diversity index. We have also confirmed the previous suggestions of N’Dri et al. (2018) that the maturity index it is a better tool of evaluating soil quality in terms of the requirements of soil fauna than indicators based only on physical and chemical criteria. However, this index requires the right choice of organisms for which it is calculated – it should be possible to establish precisely the reproductive strategies of these organisms in the examined community, and only then it should be evaluated. In our opinion, the maturity index is a reliable and helpful indicator of human-caused disturbance in soil of areas, and can therefore be useful, for example, in preparation of plans of protection of national parks and nature reserves. Finally, the index can also be used for monitoring soil quality changes in long-term research because it can be calculated on the basis of data from earlier faunistic studies.

## Supplementary Information

Below is the link to the electronic supplementary material.

Supplementary file1 (DOCX 17 kb)
