# Supplementary material for: The maturity index for Uropodina (Acari: Mesostigmata) communities as an indicator of human-caused disturbance in selected forest complexes of Poland
Source: Exp Appl Acarol. 2021 Apr 2;83(4):475–91. doi: 10.1007/s10493-021-00607-5 (PMC8041667; doi:10.1007/s10493-021-00607-5)
Supplement: Supplementary file 1 — Supplementary file1 (DOCX 17 kb) [file 10493_2021_607_MOESM1_ESM.docx]

**Supplementary materials:**

**Table S1** Species of Uropodina classified into the *r*/*K*-strategies according to the adopted criteria: D – dominance, F – frequency in samples, H – ecological tolerance, R – population growth rate, Ph – phoresy, L – occurrence of larvae per year.

| Species | D | F | H | R | Ph | L | Sum | Strategy |
| --- | --- | --- | --- | --- | --- | --- | --- | --- |
| *Metagynella paradoxa* Berlese, 1919 | 5 | 5 | 4 | 1 | 1 | 2 | 18 | *K*3 |
| *Trichouropoda bipilis* (Vitzthum 1921) | 5 | 5 | 4 | 1 | 1 | 2 | 18 | *K*3 |
| *Dinychus septentrionalis* (Trägårdh, 1943) | 5 | 5 | 4 | 1 | 0 | 2 | 17 | *K2* |
| *Oodinychus spatulifera* (Moniez, 1892) | 5 | 5 | 4 | 1 | 0 | 2 | 17 | *K*2 |
| *Oplitis minutissima* (Berlese, 1903) | 5 | 5 | 4 | 1 | 0 | 2 | 17 | *K*2 |
| *Phaulodiaspis advena* (Trägårdh, 1922) | 5 | 5 | 4 | 1 | 0 | 2 | 17 | *K*2 |
| *Phaulodiaspis rackei* (Oudemans, 1912) | 5 | 5 | 4 | 1 | 0 | 2 | 17 | *K*2 |
| *Trichouropoda polytricha* (Vitzthum, 1923) | 5 | 5 | 4 | 1 | 0* | 2 | 17 | *K*2 |
| *Trichouropoda obscura* (C.L. Koch, 1836) | 5 | 5 | 4 | 1 | 0 | 2 | 17 | *K*2 |
| *Trachyuropoda coccinea* (Michael, 1891) | 5 | 5 | 4 | 1 | 0 | 2 | 17 | *K*2 |
| *Trichouropoda patavina* (G. Canestrini, 1885) | 5 | 5 | 4 | 1 | 0 | 2 | 17 | *K*2 |
| *Uroobovella nova* (Oudemans, 1902) | 5 | 5 | 3 | 1 | 1 | 2 | 17 | *K*2 |
| *Uropolyaspis hamulifera* Berlese, 1904 | 5 | 5 | 4 | 1 | 0 | 2 | 17 | *K2* |
| *Oplitis alophora* (Berlese, 1903) | 5 | 5 | 4 | 0 | 0 | 2 | 16 | *K*1 |
| *Oplitis franzi* Hirschmann et Zirngiebl-Nicol, 1969 | 5 | 5 | 4 | 0 | 0 | 2 | 16 | *K1* |
| *Oplitis philocenta* (Trouessart, 1902) | 5 | 5 | 4 | 0 | 0 | 2 | 16 | *K*1 |
| *Oplitis schmitzi* (Kneissl, 1908) | 5 | 5 | 4 | 0 | 0 | 2 | 16 | *K*1 |
| *Oplitis stammeri* Hirschmann et Zirngiebl-Nicol, 1961 | 5 | 5 | 4 | 0 | 0 | 2 | 16 | *K*1 |
| *Oplitis wasmanni* (Kneissl, 1907) | 5 | 5 | 4 | 0 | 0 | 2 | 16 | *K*1 |
| *Phaulodinychus copridis* (Oudemans, 1916) | 5 | 5 | 4 | 0 | 0 | 2 | 16 | *K*1 |
| *Phaulodinychus spinosula* (Kneissl, 1916) | 5 | 5 | 4 | 0 | 0 | 2 | 16 | *K*1 |
| *Polyaspinus schweizeri* Hutu, 1976 | 5 | 5 | 4 | 0 | 0 | 2 | 16 | *K*1 |
| *Protodinychus punctatus* Evans, 1957 | 5 | 5 | 4 | 0 | 0 | 2 | 16 | *K*1 |
| *Trachytes splendida* Hutu, 1973 | 5 | 5 | 4 | 0 | 0 | 2 | 16 | *K*1 |
| *Trachyuropoda poppi* Hirschmann et Z.-Nicol, 1969 | 5 | 5 | 4 | 0 | 0 | 2 | 16 | *K*1 |
| *Trachyuropoda wasmanniana* Berlese, 1903 | 5 | 5 | 4 | 0 | 0 | 2 | 16 | *K*1 |
| *Trachyuropoda willmanni* Hirschmann et Z.-Nicol, 1969 | 5 | 5 | 4 | 0 | 0 | 2 | 16 | *K*1 |
| *Trichouropoda dalarnaensis* Hirschmann et Zirngiebl-Nicol, 1961 | 5 | 5 | 4 | 0 | 0 | 2 | 16 | *K*1 |
| *Trichouropoda dialveolata* Hirschmann et Zirngiebl-Nicol, 1961 | 5 | 5 | 4 | 0 | 0 | 2 | 16 | *K*1 |
| *Trichouropoda longiovalis* Hirschmann et Zirngiebl-Nicol, 1961 | 5 | 5 | 4 | 0 | 0 | 2 | 16 | *K*1 |
| *Uroobovella ipidis* (Vitzthum, 1923) | 5 | 5 | 4 | 0 | 0 | 2 | 16 | *K*1 |
| *Uropoda italica* Hirschmann et Z.-Nicol, 1969 | 5 | 5 | 4 | 0 | 0 | 2 | 16 | *K*1 |
| *Uropoda undulata* Hirschmann et Z.-Nicol, 1969 | 5 | 5 | 4 | 0 | 0 | 2 | 16 | *K*1 |
| *Uroseius hunzikeri* Schweizer, 1922 | 5 | 5 | 4 | 0 | 0 | 2 | 16 | *K1* |
| *Allodinychus flagelliger* (Berlese, 1910) | 5 | 5 | 3 | 1 | 0 | 2 | 16 | *K*1 |
| *Cilliba selnicki* (Hirschmann et Z.-Nicol, 1969) | 5 | 5 | 3 | 1 | 0 | 2 | 16 | *K*1 |
| *Dinychura cordieri* (Berlese, 1916) | 5 | 5 | 3 | 1 | 0 | 2 | 16 | *K*1 |
| *Dinychus woelkiei* Hirschmann et Zirngiebl-Nicol, 1969 | 5 | 5 | 3 | 1 | 0 | 2 | 16 | *K*1 |
| *Iphiduropoda penicillata* (Hirschmann et Z.-Nicol, 1961) | 5 | 5 | 4 | 0 | 0 | 2 | 16 | *K*1 |
| *Nenteria floralis* Kardg, 1986 | 5 | 5 | 3 | 1 | 0 | 2 | 16 | *K*1 |
| *Nenteria pandioni* Wiśniewski et Hirschmann, 1985 | 5 | 5 | 3 | 1 | 0 | 2 | 16 | *K*1 |
| *Oodinychus obscurasimilis* (Hirschmann et Z.-Nicol, 1961) | 5 | 5 | 3 | 1 | 0 | 2 | 16 | *K*1 |
| *Polyaspis sansonei* Berlese, 1916 | 5 | 5 | 3 | 1 | 0 | 2 | 16 | *K*1 |
| *Trichouropoda calcarata* (Hirschmann et Z.-Nicol, 1961) | 5 | 5 | 3 | 1 | 0 | 2 | 16 | *K*1 |
| *Uroobovella marginata* (C. L. Koch, 1829) | 5 | 5 | 3 | 1 | 0 | 2 | 16 | *K*1 |
| *Uroobovella obovata* (Canestrini et Berlese, 1884) | 5 | 5 | 3 | 1 | 0 | 2 | 16 | *K*1 |
| *Uroplitella conspicua* Berlese, 1903 | 5 | 5 | 3 | 1 | 0 | 2 | 16 | *K*1 |
| *Uroplitella paradoxa* (Canestrini et Berlese, 1884) | 5 | 5 | 3 | 1 | 0 | 2 | 16 | *K*1 |
| *Urotrachytes formicarius* (Lubbock, 1881) | 5 | 5 | 3 | 1 | 0 | 2 | 16 | *K*1 |
| *Dinychus inermis* (C. L. Koch, 1841) | 5 | 5 | 3 | 1 | 0 | 2 | 16 | *K*1 |
| *Polyaspis patavinus* Berlese, 1881 | 5 | 5 | 3 | 1 | 0* | 2 | 16 | *K*1 |
| *Trachytes minima* Trägårdh , 1910 | 5 | 5 | 3 | 1 | 0 | 2 | 16 | *K*1 |
| *Uroobovella baloghi* Hirschmann et Zirngiebl-Nicol, 1962 | 5 | 5 | 3 | 1 | 0 | 2 | 16 | *K*1 |
| *Fuscouropoda appendiculata* (Berlese, 1910) | 5 | 5 | 3 | 1 | 0 | 2 | 16 | *K*1 |
| *Trichouropoda structura* (Hirschmann et Z.-Nicol, 1961) | 5 | 5 | 3 | 1 | 0 | 2 | 16 | *K*1 |
| *Trichouropoda tuberosa* (Hirschmann et Z.-Nicol, 1961) | 5 | 5 | 3 | 1 | 0 | 2 | 16 | *K*1 |
| *Uroobovella fimicola* (Berlese, 1903) | 5 | 5 | 3 | 1 | 0 | 2 | 16 | *K*1 |
| *Uroobovella fracta* (Berlese, 1916) | 5 | 5 | 3 | 1 | 0 | 2 | 16 | *K*1 |
| *Uroobovella vinicolora* (Vitzthum, 1926) | 5 | 5 | 3 | 1 | 0 | 2 | 16 | *K*1 |
| *Cilliba insularis* Willmann, 1938 | 5 | 5 | 3 | 1 | 0 | 2 | 16 | *K*1 |
| *Olodiscus kargi* (Hirschamann et Z.-Nicol, 1969) | 5 | 5 | 3 | 1 | 0 | 2 | 16 | *K*1 |
| *Uropoda fumicola* Hirschmann et Z.-Nicol, 1969 | 5 | 5 | 3 | 1 | 0 | 2 | 16 | *K*1 |
| *Uroseius geieri* (Schweizer, 1961) | 5 | 5 | 4 | 0 | 0 | 2 | 16 | *K*1 |
| *Trematurella elegans* (Kramer, 1882) | 5 | 5 | 3 | 1 | 0 | 2 | 16 | *K*1 |
| *Discourella baloghi* Hirschmann et Z.-Nicol, 1969 | 5 | 5 | 3 | 1 | 0 | 2 | 16 | *K*1 |
| *Trichouropoda sociata* (Vitzthum, 1923) | 5 | 5 | 3 | 1 | 0* | 2 | 16 | *K*1 |
| *Apionoseius infirmus* Berlese, 1887 | 4 | 5 | 4 | 1 | 0* | 2 | 16 | *K*1 |
| *Trachytes lamda* Berlese, 1903 | 5 | 5 | 4 | 0 | 0 | 2 | 16 | *K*1 |
| *Cilliba cassidea* (Herman, 1804) | 5 | 5 | 2 | 1 | 0 | 2 | 15 | *r*3 |
| *Dinychus arcuatus* (Trägårdh, 1922) | 5 | 5 | 2 | 1 | 0 | 2 | 15 | *r*3 |
| *Urodiaspis stammeri* Hirschmann et Z.-Nicol, 1969 | 5 | 5 | 2 | 1 | 0 | 2 | 15 | *r*3 |
| *Discourella modesta* (Leonardi, 1889) | 5 | 5 | 3 | 0 | 0 | 2 | 15 | r3 |
| *Nenteria stylifera* (Berlese, 1904) | 5 | 5 | 3 | 0 | 0 | 2 | 15 | *r*3 |
| *Trachytes montana* Willmann, 1953 | 5 | 5 | 3 | 0 | 0 | 2 | 15 | *r*3 |
| *Nenteria breviunguiculata* (Willmann, 1949) | 4 | 5 | 3 | 1 | 0 | 2 | 15 | *r*3 |
| *Leiodinychus orbicularis* (C. L. Koch, 1839) | 3 | 5 | 4 | 1 | 0* | 2 | 15 | *r*3 |
| *Pulchellaobovella pyriformis* (Berlese, 1920) | 4 | 5 | 2 | 1 | 1 | 2 | 15 | *r*3 |
| *Phaulodiaspis borealis* Sellnick, 1940 | 3 | 5 | 4 | 1 | 0* | 2 | 15 | *r*3 |
| *Cilliba rafalskii* (Błoszyk Stachowiak et Halliday, 2008) | 5 | 5 | 5 | 0 | 0 | 0 | 15 | *r*3 |
| *Olodiscus misella* (Berlese, 1916) | 5 | 5 | 3 | 0 | 0 | 1 | 14 | *r*2 |
| *Cilliba cassideasimilis* (Błoszyk Stachowiak et Halliday, 2008) | 5 | 5 | 2 | 1 | 0 | 1 | 14 | *r*2 |
| *Dinychus carinatus* Berlese, 1903 | 4 | 5 | 3 | 1 | 0 | 1 | 14 | *r*2 |
| *Cilliba erlangensis* (Hirschmann et Z.-Nicol, 1969) | 5 | 5 | 2 | 0 | 0 | 2 | 14 | *r*2 |
| *Uropoda orbicularis* (Muller, 1776) | 5 | 5 | 2 | 0 | 0* | 2 | 14 | *r*2 |
| *Neodiscopoma splendida* (Kramer, 1882) | 4 | 5 | 2 | 1 | 0 | 2 | 14 | *r*2 |
| *Polyaspinus cylindricus* Berlese, 1916 | 5 | 5 | 2 | 1 | 0 | 0 | 13 | *r*2 |
| *Pulchellaobovella pulchella* (Berlese, 1904) | 3 | 5 | 3 | 0 | 0* | 0 | 11 | *r*2 |
| *Dinychus perforatus* Kramer, 1882 | 3 | 4 | 2 | 0 | 0 | 1 | 10 | *r1* |
| *Urodiaspis pannonica* Willmann, 1952 | 4 | 4 | 2 | 0 | 0 | 0 | 10 | *r*1 |
| *Trachytes irenae* Pecina, 1970 | 2 | 4 | 1 | 0 | 0 | 1 | 8 | *r*1 |
| *Oodinychus karawaiewi* (Berlese, 1903 ) | 2 | 4 | 1 | 0 | 0 | 0 | 7 | *r*1 |
| *Trachytes pauperior* (Berlese, 1914) | 3 | 3 | 1 | 0 | 0 | 0 | 7 | *r*1 |
| *Urodiaspis tecta* (Kramer, 1876) | 2 | 3 | 2 | 0 | 0 | 0 | 7 | *r*1 |
| *Oodinychus ovalis* (C. L. Koch, 1839) | 1 | 3 | 1 | 0 | 0* | 0 | 5 | *r*1 |
| *Olodiscus minima* (Kramer, 1882) | 1 | 2 | 1 | 0 | 0 | 0 | 4 | *r*1 |
| *Trachytes aegrota* (C. L. Koch, 1841) | 1 | 1 | 1 | 0 | 0 | 0 | 3 | *r*1 |

*Footnote*: *phoretic species with a lot of deutonymphs carried (effective phoresy).

**Table S2** Assemblages of Uropodina inhabiting examined areas with *r*/*K*-strategy classification.

|  | Cisy Staropolskie im. Leona Wyczółkowskiego NR | Białowieża Primeval Forest | Gorce NP | Jakubowo NR | Las Grądowy NR |
| --- | --- | --- | --- | --- | --- |
| *O. minima* | *r*1 | *r*1 | *r*1 | *r*1 | *r*1 |
| *O. ovalis* | *r*1 | *r*1 | *r*1 | *r*1 | *r*1 |
| *T. aegrota* | *r*1 | *r*1 | *r*1 | *r*1 | *r*1 |
| *T. pauperior* | *r*1 | *r*1 | *r*1 | *r*1 | *r*1 |
| *U. tecta* | *r*1 | *r*1 | *r*1 | *r*1 | *r*1 |
| *C. cassideasimilis* | *r*2 |  | *r*2 | *r*2 | *r*2 |
| *U. pannonica* | *r*1 | *r*1 |  | *r*1 | *r*1 |
| *D. perforatus* | *r*1 | *r*1 | *r*1 |  | *r*1 |
| *D. arcuatus* | *r*3 | *r*3 | *r*3 |  |  |
| *D. carinatus* | *r*2 | *r*2 | *r*2 |  |  |
| *N. splendida* | *r2* | *r2* | *r2* |  |  |
| *O. kargi* | *K*1 | *K*1 |  | *K*1 |  |
| *O. misella* | *r*2 | *r*2 | *r*2 |  |  |
| *P. cylindricus* |  |  | *r*2 | *r*2 | *r*2 |
| *P. rackei* | *K*2 |  |  | *K*2 | *K*2 |
| *T. lamda* | *K*1 |  |  | *K*1 | *K*1 |
| *P. pulchella* | ***r***2 | *r*2 | *r*2 |  |  |
| *D. inermis* | *K*1 |  |  | *K*1 |  |
| *D. woelkei* | *K*1 |  |  |  |  |
| *D. modesta* | *r*3 | *r*3 |  |  |  |
| *L. orbicularis* | *r*3 | *r*3 |  |  |  |
| *O. obscurasimilis* | *K*1 |  | *K*1 |  |  |
| *O. karawaiewi* | *r*1 | *r*1 |  |  |  |
| *Oplitis sp.* | *K*2 | *K*2 |  |  |  |
| *T. calcarata* |  |  | *K*1 | *K*1 |  |
| *Pseudouropoda ssp.* | *K*1 |  |  |  |  |
| *T. elegans* | *K*1 | *K*1 |  |  |  |
| *C. rafalskii* | *r*3 |  |  |  |  |
| *C. insularis* |  | *K*1 |  |  |  |
| *D. cordieri* | *K*1 |  |  |  |  |
| *D. septentrionalis* |  | *K*2 |  |  |  |
| *I. penicillata* | *K*1 |  |  |  |  |
| *N. breviunguiculata* |  |  | *r*3 |  |  |
| *N. stylifera* |  |  |  | *r*3 |  |
| *P. patavinus* | *K*1 |  |  |  |  |
| *P. sansonei* |  | *K*1 |  |  |  |
| *T. irenae* |  |  | *r*1 |  |  |
| *T. minima* |  |  | *K*1 |  |  |
| *U. obovata* | *K*1 |  |  |  |  |
| *U. orbicularis* | *r*2 |  |  |  |  |
| *P. pyriformis* |  |  |  | *r*3 |  |
| *U. fumicola* |  | *K*1 |  |  |  |
| *U. hamulifera* |  | *K*2 |  |  |  |
| Total numer of species | **31** | **23** | **18** | **15** | **11** |
| % of 43 found taxa | 72.09 | 53.49 | 41.86 | 34.88 | 25.58 |
| Number of *K*-species | 13 | 8 | 3 | 5 | 2 |
| Number of *r*-species | 18 | 15 | 15 | 10 | 9 |
